# Neuroprotective Effects of Salidroside in the MPTP Mouse Model of Parkinson's Disease: Involvement of the PI3K/Akt/GSK3*β* Pathway

**DOI:** 10.1155/2016/9450137

**Published:** 2016-09-21

**Authors:** Wei Zhang, Hong He, Hujie Song, Junjie Zhao, Tao Li, Leitao Wu, Xiaojun Zhang, Jianzong Chen

**Affiliations:** ^1^Research Center of Traditional Chinese Medicine, Xijing Hospital, Fourth Military Medical University, Xi'an 710032, China; ^2^Department of Encephalopathy, Xi'an Encephalopathy Hospital of Traditional Chinese Medicine, Xi'an 710032, China; ^3^Department of Physics, Fourth Military Medical University, Xi'an 710032, China

## Abstract

The degenerative loss through apoptosis of dopaminergic neurons in the substantia nigra pars compacta plays a primary role in the progression of Parkinson's disease (PD). Our in vitro experiments suggested that salidroside (Sal) could protect against 1-methyl-4-phenylpyridine-induced cell apoptosis in part by regulating the PI3K/Akt/GSK3*β* pathway. The current study aims to increase our understanding of the protective mechanisms of Sal in the 1-methyl-4-phenyl-1,2,3,6-tetrahydropypridine- (MPTP-) induced PD mouse model. We found that pretreatment with Sal could protect against MPTP-induced increase of the time of turning downwards and climbing down to the floor. Sal also prevented MPTP-induced decrease of locomotion frequency and the increase of the immobile time. Sal provided a protection of in MPTP-induced loss of tyrosine hydroxylase-positive neurons in SNpc and the level of DA, DOPAC, and HVA in the striatum. Furthermore, Sal could increase the phosphorylation level of Akt and GSK3*β*, upregulate the ratio of Bcl-2/Bax, and inhibit the activation of caspase-3, caspase-6, and caspase-9. These results show that Sal prevents the loss of dopaminergic neurons and the PI3K/Akt/GSK3*β* pathway signaling pathway may have mediated the protection of Sal against MPTP, suggesting that Sal may be a potential candidate in neuroprotective treatment for PD.

## 1. Introduction

Parkinson's disease (PD), as one of the most common neurodegenerative disorders, displays characteristic motor and behavioral disturbances, including static tremor, rigidity, bradykinesia, and posture gait disorders. It is primarily caused by the degenerative deletion through apoptosis of dopaminergic (DA) neurons in the substantia nigra pars compacta (SNpc) [1]. Therapeutic approaches including numerous drugs have been greatly identified, but most of drugs which were explored for clinical approval cannot suspend or stop the progression of PD with inevitable obvious adverse effects [2–5]. Although its pathogenesis remains unclear, current evident indicates that Akt, also known as protein kinase B, plays a potential mechanistic role of defective signaling in PD [6–8]. It hypothesizes that pharmacological compounds which recover the defective Akt activity might be an operative method for striking neurotrophic and antiapoptotic effects.

The phosphoinositide 3-kinase (PI3K)/Akt pathway is a critical pathway related to survival, proliferation, and growth in response to extracellular signals in neurons [9–11]. PI3K phosphorylates the 3-position hydroxyl group in the inositol ring of phosphoinositides and then recruits Akt contained pleckstrin homology domain to translocate to the plasma membrane. As a serine/threonine-specific protein kinase, it has been found that Akt and phosphorylated Akt have a marked decrease in the SNpc of PD patients [6]. In addition, the active PI3K/Akt pathway increases the survival and growth of DA neuronal cells by inhibiting apoptosis [12–14]. Glycogen synthase kinase-3 (GSK-3), as one of the substrates of Akt, is a pleiotropic serine/threonine protein kinase [15, 16]. It contains two isoforms of GSK-3*α* and GSK-3*β*. Some evidences discovered that Akt could inhibit the activity of GSK3 by phosphorylating at Ser21 in GSK-3*α* or Ser9 in GSK-3*β* [15, 17]. GSK-3*β* dysregulation results in Parkinson's-like pathophysiology; meanwhile, activation of GSK-3*β* has been shown to facilitate numerous apoptotic conditions in PD [18, 19].

Salidroside (Sal, 2-(4-hydroxyphenyl)ethyl *β*-D-glucopyranoside, C_14_H_20_O_7_) is mainly extracted from* Rhodiola rosea *L., which grows at high altitude localities and usually is used as one of the herbal drugs in worldwide [20]. Sal has been shown to have numerous pharmacological effects, such as antioxidant effects [21, 22], antiapoptosis effects [23], and maintenance of mitochondrial function [24]. Our paper recently found that Sal protected DA neurons against 1-methyl-4-phenyl-1,2,3,6-tetrahydropyridine- (MPTP-)/1-methyl-4-phenyl-pyridinium- (MPP^+^-) induced toxicity in a dose-dependent manner through modulation of the ROS-NO related mitochondrial pathway both in vivo and in vitro [25]. Moreover, we also have shown that Sal could protect against MPP^+^-induced apoptosis through modulation of the PI3K/Akt pathway in vitro [26]. Based on the result, this study aimed to further evaluate the neuroprotective effects of Sal in the MPTP-induced PD mouse model and determine whether its protective mechanisms relate to the PI3K/Akt/GSK3*β* pathway, so that it can provide evidence for Sal as a potential target for effective neuroprotective treatment for PD.

## 2. Materials and Methods 

### 2.1. Animals and Treatment

C57BL/6 mice (male, eight weeks old, weighing 23–28 g) were supplied by the Experimental Animal Center of the Fourth Military Medical University and fed in a 12 h on/off light cycle in a temperature-controlled room (23 ± 1°C). All mice were reared in a single cage with food and water provided ad libitum for 7 days before the start of experiments. All procedures were approved by the Animal Care and Use Committee of the Fourth Military Medical University.

The subacute MPTP mice model was carried out depending on the previously published methods [27]. All the mice were divided by the random number method into 5 groups (*n* = 10 per group): control group, in which mice were intraperitoneally injected with saline (30 mg/kg/day) for 12 days, Sal (HPLC ≥98%, National Institute for the Control of Pharmaceutical and Biological Products, Xi'an, China) group, in which mice were intraperitoneally injected with Sal (45 mg/kg/day) for 12 days, MPTP (Sigma-Aldrich, MO, USA) group, in which mice were intraperitoneally injected with saline (30 mg/kg/day) for 7 days and then MPTP (30 mg/kg/day) for 5 consecutive days, Sal + MPTP group I, in which mice were intraperitoneally injected with Sal (15 mg/kg/day) for 7 days and then MPTP (30 mg/kg/day) for 5 consecutive days, and Sal + MPTP group II, in which mice were intraperitoneally injected with Sal (45 mg/kg/day) for 7 days and then MPTP (30 mg/kg/day) for 5 consecutive days. After the last treatment at 24 h, the mice were managed to subsequent tests.

### 2.2. Pole Test

Pole tests were performed following a previously published protocol starting on the 1st day after treatment began [28]. Mice were permitted to adapt to the experimental environment for 2 days before the first test. Each mouse was placed on the top of a pole with a rough surface (1 cm in diameter and 55 cm in height) with its head facing upwards. The time in which the mouse completely turned downwards (T-turn) and climbed down to the floor (T-LA) was recorded.

### 2.3. Open Field Test

The open field test evaluated the change of locomotory capacity according to the previously published protocol [29]. A wooden box (40 × 40 × 40 cm^3^) was horizontally divided into 16 squares of equal size (10 × 10 cm^2^). The central 4 squares (20 × 20 cm^2^) were considered the center, and the surrounding 4 sides (10 × 10 cm^2^) and 4 corners (10 × 10 cm^2^) were considered the periphery. After a mouse was put in the center of the box, a 30 min test session was initiated. The dynamic activity of mice was recorded by an automatic video tracking system (Shanghai Jiliang Software Technology Co., Ltd., Shanghai, China). The percentage of speed and time spent in the center of the arena was used to assess the motor and behavioral changes.

### 2.4. Immunofluorescence Staining

Immunofluorescence histochemistry of mouse brain tissue was performed according to previously published protocols [30]. The tissue was cut into 30 *μ*m slices with a sliding microtome. In the staining experiments, it was fixed in 4% paraformaldehyde for 15 min followed by incubation in 0.2% Triton X-100 permeabilization solution for 1 h and 1% bovine serum albumin for 2 h. Sections were then incubated in a chicken anti-mouse tyrosine hydroxylase (TH) antibody (Abcam, CA, USA) diluted at 1 : 200 in primary antibody dilution buffer at 4°C overnight. After rinsing three times in PBS for 5 minutes, slides were incubated with Alexa Fluor® 594 Goat Anti-Chicken IgG secondary antibody (Life Technologies, NY, USA) for 2 h at room temperature in the dark. Cover slips were mounted onto slides with 0.5% glycerin. The section was analyzed on an immunofluorescence microscopy (IX51, Olympus, Japan). The TH-positive neuron cells in 5 position-matched sections of each mouse were manually counted by ImageJ software (version 1.48) by a technician who was blinded to this study [31]. The average number of immunoreactive neurons per section represents the livability.

### 2.5. High Performance Liquid Chromatography (HPLC) Analysis of Striatal Dopamine and Its Metabolite Level

High performance liquid chromatography (HPLC) was used to analyze the dopamine (DA) and its metabolites, including dihydroxy-phenyl acetic acid (DOPAC) and homovanillic acid (HVA), in the striatum, as previously described [32]. The striatum was rapidly isolated and was homogenized by 100 *μ*L ice-cold 0.2 N perchloric acid. After the homogenization, it was centrifuged at 13,000 ×g for 15 min at 4°C. The supernatant was filtered by the 0.45 mm of filter and then tested by electrochemical detection (Eicom, Kyoto, Japan) for HPLC. The concentration was expressed as ng/mg tissue.

### 2.6. Western Blots

The tissue was collected and ground and combined with a cell lysate solution. Protein quantification was performed with a bicinchoninic acid protein assay kit (Life Technologies, NY, USA). In Western blot tests, after electrophoresis, the transferred PVDF membranes were incubated overnight with antibodies against Akt, phospho-Akt (Ser473), GSK-3*β*, and phospho-GSK-3*β* (Ser9) (Cell Signaling Technology, MA, USA), B-cell lymphoma-2 (Bcl-2), Bax, or cleaved cysteine-dependent aspartate-directed proteases (caspase-3, caspase-6, or caspase-9) (Abcam, CA, USA). All antibodies were diluted to 1 : 500–1 : 3000 and were incubated at 4°C. Membranes were subjected to 3 times × 5 min washes in PBS and were then incubated for 2 h with HRP-conjugated anti-rabbit IgG antibody. Protein levels were measured with Quantity One analysis software (Bio-Rad, CA, USA). All experiments were conducted in triplicate.

### 2.7. Statistical Analyses

Data were expressed as the mean ± SEM from at least three independent experiments. Statistical significance was analyzed by one-way analysis of variance (ANOVA) or Student's* t*-test in SPSS version 20.0. A value of *P* < 0.05 was considered statistically significant.

## 3. Results

### 3.1. Sal Prevents Behavioral Disorders

**Figure 1:**
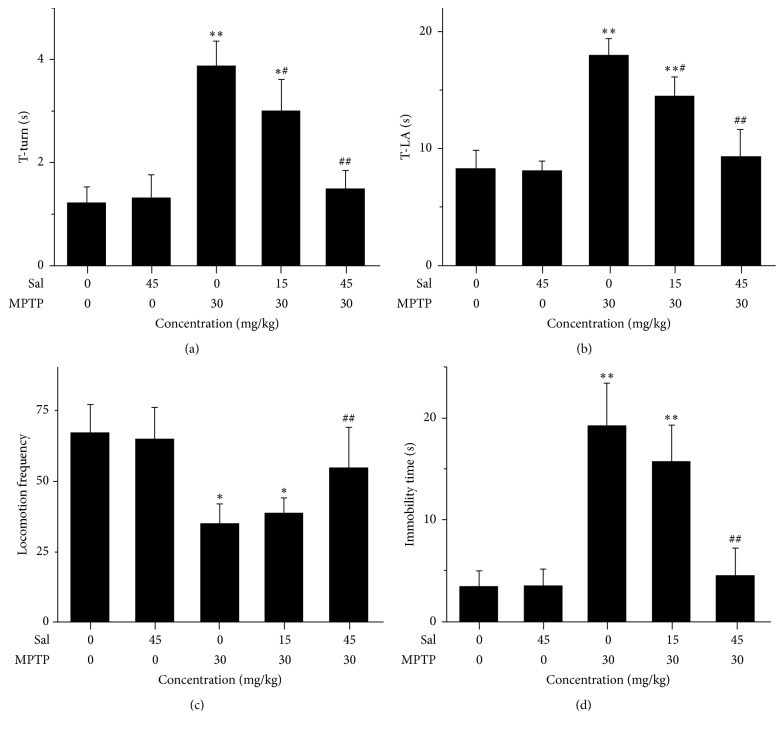
Sal improved behavior disorders in the MPTP-induced PD mouse model in pole tests and open field tests. Mice were pretreated with a sham solution or Sal (15 or 45 mg/kg). (a) Time spent to completely turn downward (T-turn). (b) Time spent getting from the top of the pole to the floor (T-LA). (c) Locomotion frequency. (d) Time spent immobile. Each column represents the mean ± SEM (*n* = 10). ^*∗*^ *P* < 0.05 and ^*∗∗*^ *P* < 0.01 compared with the control group; ^#^ *P* < 0.05 and ^##^ *P* < 0.01 compared with the MPTP group.

The protective effect of Sal against PD-related behavioral disorders was evaluated by pole test and open field test in the MPTP-induced PD mouse model. In the pole test, MPTP treatment significantly extended the time of T-turn to 3.9 ± 0.5 s (*P* < 0.01) and that of T-LA to 18.0 ± 1.4 s (*P* < 0.01) compared with the control group (Figures 1(a) and 1(b)). Pretreatment with Sal significantly blocked the increase of T-turn and T-LA time in the Sal + MPTP group I (*P* < 0.05; *P* < 0.01) and Sal + MPTP group II (*P* < 0.01; *P* < 0.01) compared with the MPTP group; however, it merely had no significant difference in Sal + MPTP group II when compared with control group (*P* > 0.05). In the open field test, the locomotion frequency significantly reduced to 35.1 ± 7.2 times (*P* < 0.05) and immobility time increased to 19.3 ± 4.2 s (*P* < 0.01) in the MPTP group when compared with the control group (Figures 1(c) and 1(d)). Pretreatment with Sal had no significant changes in either locomotion frequency or immobile time in the Sal + MPTP group I (*P* > 0.05). However, pretreatment with Sal significantly prevented the locomotion frequency (*P* < 0.01) and decreased the immobile time (*P* < 0.01) in the Sal + MPTP group II (Figures 1(c) and 1(d)), and it had no significant difference when compared with control group (*P* > 0.05).

### 3.2. Sal Prevents the Loss of TH-Positive Neurons

**Figure 2:**
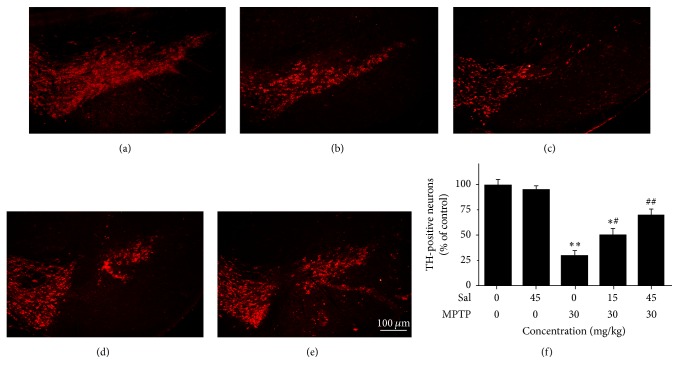
Sal protects the loss of TH-positive neurons in the SNpc in the MPTP-induced PD mouse model; (a) control group; (b) Sal group; (c) MPTP group; (d) Sal + MPTP group I; (e) Sal + MPTP group II. Bar = 100 *μ*m. (f) Histogram shows the number of TH-positive neurons in the control group as standard. Each column represents mean ± SEM (*n* = 10). ^*∗*^ *P* < 0.05 and ^*∗∗*^ *P* < 0.01 compared with the control group; ^#^ *P* < 0.05 and ^##^ *P* < 0.01 compared with the MPTP group.

There was no significant difference in the number of TH-positive neurons (100.0 ± 5.2%) in the control group and in the Sal group (95.3 ± 3.6%) (Figures 2(a), 2(b), and 2(f)). However, a significant reduction was observed in the number of TH-positive neurons in the MPTP group compared with the control group (*P* < 0.01) (Figures 2(a), 2(c), and 2(f)). Compared with MPTP group, although pretreatment with Sal significantly restored the number of TH-positive neurons in the Sal + MPTP group I (50.2 ± 6.4% versus 30.4 ± 4.2%) (*P* < 0.05), it was still lower than that in control group (*P* < 0.05). However, pretreatment with Sal could significantly restore that number to 70.2 ± 5.7% in the Sal + MPTP group II (*P* < 0.01) (Figures 2(c), 2(e), and 2(f)), and it had no significant difference when compared with control group (*P* > 0.05).

### 3.3. Sal Prevents the Loss of Striatal DA, DOPAC, and HVA Level

**Figure 3:**
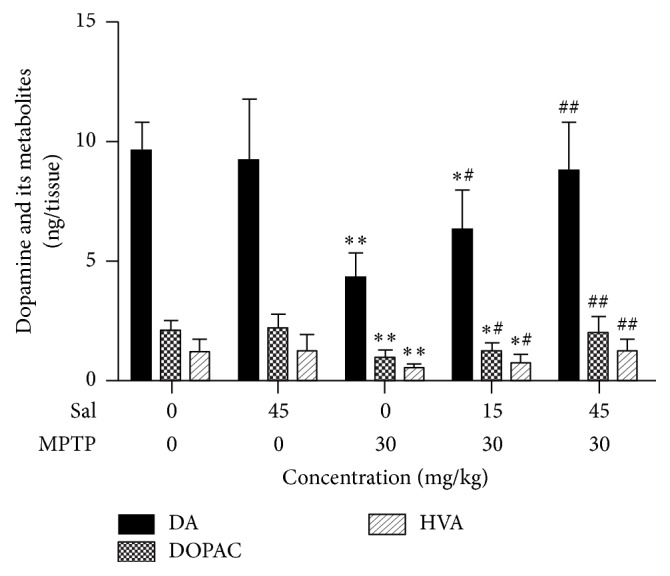
Sal increases the level of striatal DA and its metabolites in the MPTP-induced PD mouse model. DA and its metabolites (DOPAC and HVA) were measured by HPLC. Data represent three independent experiments. Each column represents the mean ± SEM (*n* = 10). ^*∗*^ *P* < 0.05 and ^*∗∗*^ *P* < 0.01 compared with the control group; ^#^ *P* < 0.05 and ^##^ *P* < 0.01 compared with the MPTP group.

DA, DOPAC, and HVA can directly express the content of dopamine in the striatum of mice. It was found that the level of DA, DOPAC, and HVA had no statistical obvious change in the Sal group, when compared with control group (Figure 3). It significantly reduced these levels in the MPTP group (*P* < 0.01); however, it statistically significantly increased all of them in a dose-dependent way in the Sal + MPTP group I (*P* < 0.05) and in the Sal + MPTP group II (*P* < 0.01). And they merely had no significant difference in the Sal + MPTP group II when compared with control group (*P* > 0.05).

### 3.4. Sal Prevents the Level of pSer473-Akt and pSer9-GSK-3*β*

**Figure 4:**
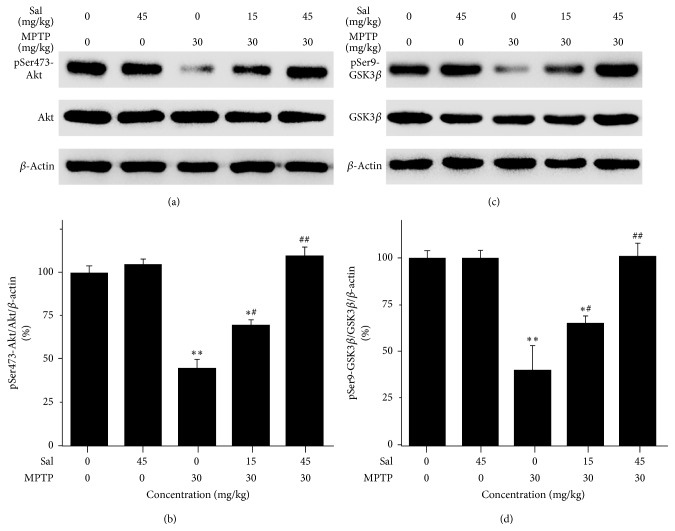
Sal increases the level of pSer473-Akt and pSer9-GSK-3*β* in the MPTP-induced PD mouse model. (a) Protein expression of pSer473-Akt and Akt. (b) Quantification is normalized to the control group of (a). Data represents three independent experiments. Each column represents the mean ± SEM (*n* = 10). (c) Protein expression of pSer9-GSK-3*β* and GSK-3*β*. (d) Quantification is normalized to the control group of (c). Data represents three independent experiments. Each column represents the mean ± SEM (*n* = 10). ^#^ *P* < 0.05 and ^##^ *P* < 0.01 compared with the MPTP group.

Next the role of Akt and GSK-3*β* in Sal protection against MPTP-induced toxicity is explored. There was no difference in the total level of Akt and GSK-3*β* among all groups (Figures 4(a) and 4(c)). MPTP treatment induced a decrease in pSer473-Akt/Akt ratio compared with the control group (45.0 ± 5.1% versus 100.0 ± 4.5%) (*P* < 0.01) (Figures 4(a) and 4(b)). Pretreatment with Sal could prevent the pSer473-Akt level to 70.0 ± 3.2% in the Sal + MPTP group I (*P* < 0.05) and 95.1 ± 5.1% in the Sal + MPTP group II (*P* < 0.01); however, it merely had no significant difference in Sal + MPTP group II when compared with control group (*P* > 0.05). In addition, MPTP treatment induced a decrease in pSer9-GSK-3*β*/GSK-3*β* ratio compared with the control group (40.5 ± 13.2% versus 100.0 ± 4.0%) (*P* < 0.01) (Figures 4(c) and 4(d)). It significantly prevented the pSer9-GSK-3*β*/GSK-3*β* level to 65.2 ± 4.1% in the Sal + MPTP group I (*P* < 0.05) and 101.1 ± 7.0% in the Sal + MPTP group II (*P* < 0.01); however, it also had no significant difference in Sal + MPTP group II when compared with control group (*P* > 0.05).

### 3.5. Sal Prevents the Ratio of Bcl-2 and Bax

**Figure 5:**
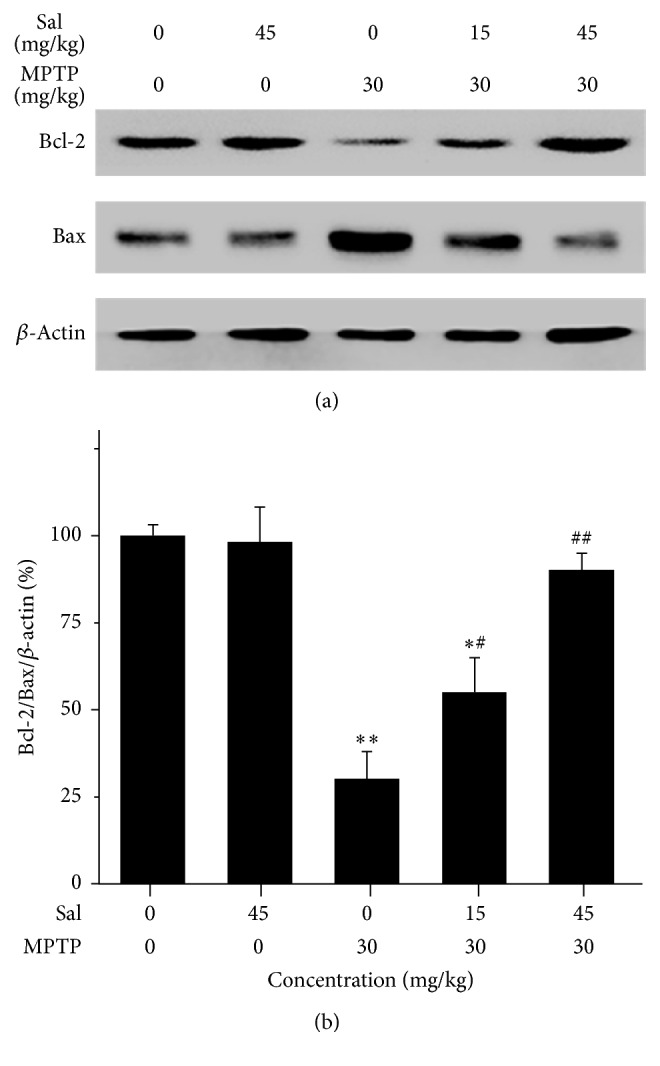
Sal upregulates the protein level of Bcl-2 and downregulates the protein level of Bax in the MPTP-induced PD mouse model. (a) Protein expression of Bcl-2 and Bax. (b) Quantification is normalized to the control group. Data represent three independent experiments. Each column represents the mean ± SEM (*n* = 10). ^*∗*^ *P* < 0.05 and ^*∗∗*^ *P* < 0.01 compared with control group; ^#^ *P* < 0.05 and ^##^ *P* < 0.01 compared with MPTP group.

The balance of the Bcl-2/Bax ratio is an indicator of the activation of proapoptotic signaling and is related to cell survival or death. It investigated whether treatment with Sal protected against MPTP-induced toxicity involving Bcl-2 and Bax in vivo. It significantly reduced the Bcl-2/Bax ratio of 31.2 ± 8.0% in the MPTP Group (*P* < 0.01) (Figures 5(a) and 5(b)). Pretreatment with Sal significantly restored the Bcl-2/Bax ratio to 55.5 ± 8.3% in the Sal + MPTP group I (*P* < 0.05) and 90.4 ± 9.1% in the Sal + MPTP group II (*P* < 0.01); however, it merely had no significant difference in the Sal + MPTP group II when compared with control group (*P* > 0.05).

### 3.6. Sal Inhibits the Cleavage of Caspase-3, Caspase-6, and Caspase-9

**Figure 6:**
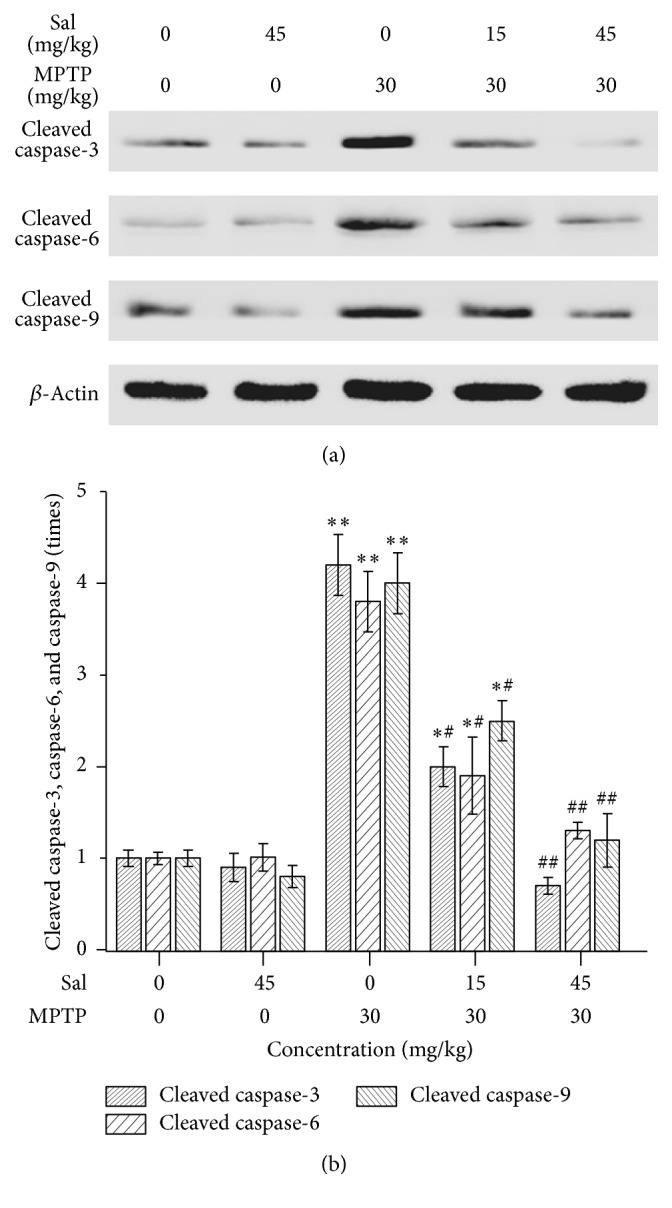
Sal inhibits the cleavage of caspase-3, caspase-6, and caspase-9 in the MPTP-induced PD mouse model. (a) Protein expression of cleaved caspase-3, caspase-6, and caspase-9. (b) Quantification is normalized to the control group. Data represent three independent experiments. Each column represents the mean ± SEM (*n* = 10). ^*∗*^ *P* < 0.05 and ^*∗∗*^ *P* < 0.01 compared with the control group; ^#^ *P* < 0.05 and ^##^ *P* < 0.01 compared with the MPTP group.

Caspase family members are key mediators that activate apoptotic pathways. Western blot showed that MPTP significantly increased the cleaved level of caspase-3 to 4.2 ± 0.3-fold, caspase-6 to 3.8 ± 0.3-fold, and caspase-9 to 4.0 ± 0.5-fold in the MPTP group compared with the control group (*P* < 0.01) (Figures 6(a) and 6(b)). Compared with the MPTP group, although pretreatment with Sal significantly decreased the cleavage of these caspases to 2.0 ± 0.2-, 1.9 ± 0.4-, and 2.5 ± 0.2-fold, respectively, in the Sal + MPTP group I (*P* < 0.05), the cleavages were still higher than those in control group (*P* < 0.05). Pretreatment with Sal significantly reduced caspases cleavage in the Sal + MPTP group II compared with the MPTP group (*P* < 0.01); and the cleavages had no significant difference when compared with control group (*P* > 0.05).

## 4. Discussion

The present study shows that pretreatment with Sal prevents the behavioral disorders and the reduced numbers of TH-positive neurons in the SNpc and the level of DA, DOPAC, and HVA in the striatum in the MPTP-induced PD mouse model. Sal protects against MPTP-induced toxicity in part through the regulation of the PI3K/Akt/GSK3*β* signaling pathway, the upregulation of Bcl-2/Bax ratio, and the inhibition of the cleavage of caspase-3, caspase-6, and caspase-9.

MPTP, as a selective toxin for DA neurons, can cause Parkinsonism which has been generally used for PD models in vivo [33, 34]. Postmortem brain biochemistry has revealed that the movement disorders associated with PD are caused by the degeneration of DA neurons in SNpc [35]. By appearance of PD symptoms, it was found that at least 50% of all nigral neurons have degenerated in SNpc, and 80% of DA levels were depleted in striatum [36–38]. In this study, pretreatment with the higher concentration of Sal (45 mg/kg) significantly blocked the increase of the time of T-turn and T-LA compared with the MPTP group. It also prevented the locomotion frequency and decreased the immobile time.

The obvious loss of dopamine neurons in the SN is one of the typical pathological characteristics of PD. The nigrostriatal pathway as one of the four major DA pathways in the brain is particularly involved in the production of DA. TH plays a key regulatory role in cellular responses to changes in the rates of biosynthesis and release of catecholamines (DA, norepinephrine, and epinephrine) [2, 39]. Studies have shown that the presence of missense mutations in TH on both alleles could cause severe Parkinsonian-related phenotypes [40]. It was further found that kind of TH-deficient mice would die at an early age [3, 41]. In this study, MPTP-treated mice showed a 30.4% of remaining in TH-positive neurons, which is consistent with previous reports [37, 38]. However, 45 mg/kg of Sal provided the protection of TH-positive neurons in the higher concentration which compared with 15 mg/kg of Sal. In addition, it was also found that Sal (45 mg/kg) prevented the loss of striatal DA, DOPAC, and HVA level in the MPTP-induced mice without influencing the normal level of them in the Sal Group. These illustrate that Sal plays the important protective effect in order to prevent the reduced number of DA neurons and the level of DA and its metabolites in the nigrostriatal pathway in MPTP-induced mice.

PI3K/Akt pathway is one of critical components of cell survival, proliferation, and growth pathways in many different cell types including neurons [8, 42]. Akt, as the substrate of PI3K, plays a primary role in the antiapoptotic pathway [8, 42]. Active PI3K/Akt signaling pathway can prevent DA neurons loss in MPTP or MPTP-like neurotoxins [43, 44]. Many pharmacological compounds have been illustrated to exert their neuroprotective effects against oxidative stress through activating the Akt pathway [45–48]. Moreover, stress stimulation could induce the translocation of Akt to mitochondria for phosphorylation, followed by the rapid inhibition of mitochondrial GSK-3*β* by phosphorylation at Ser9 [49]. The phosphorylation of GSK-3*β*, a determining factor for apoptosis, has been shown to result in neuronal cell death [13, 50, 51]. In this study, it was shown that MPTP treatment induced a decrease in pSer473-Akt/Akt level and pSer9-GSK-3*β*/GSK-3*β* level. Only the higher level of Sal (45 mg/kg) could prevent their level. Hence, combining our present study and previous studies [26], it is demonstrated that pretreatment with Sal could increase the level of pSer473-Akt and pSer9-GSK-3*β* both in vitro and in vivo.

Bcl-2 family members, as antiapoptotic or proapoptotic regulators, play a wide role in cellular activities [52]. The ratio of Bcl-2/Bax, as an indicator of the activation of proapoptotic signaling, is related to cell survival or death [19]. Akt upregulates the expression of Bcl-2 and thus leads to cell survival by phosphorylating and inhibiting proapoptotic proteins such as Bax [14, 53, 54]. In this study, treatment with Sal (45 mg/kg) significantly inhibited the MPTP-induced decrease of Bcl-2 level and increase of Bax level. Caspases are essential in apoptosis [55]. Akt phosphorylates caspase-9 promoting the cell survival [14]. It also phosphorylates caspase-3 and caspase-6, thus inhibiting their proapoptotic functions in a variety of cell lines [42, 50, 51]. In this study, pretreatment with Sal (45 mg/kg) significantly decreased the cleavage of caspase-3, caspase-6, and caspase-9 compared with the MPTP group. It was indicated that pretreatment with Sal by an appropriate concentration could increase the antiapoptosis protein and decrease the proapoptosis protein for promoting the cell survival.

## 5. Conclusion

In conclusion, Sal not only prevents the behavioral disorders but also inhibits the reduced numbers of TH-positive neurons and the level of DA, DOPAC, and HVA in the MPTP-induced PD mouse model. This closely related with the regulation of the PI3K/Akt/GSK3*β* signaling pathway, the upregulation of a normal Bcl-2/Bax ratio, and the inhibition of the cleaved caspase-3, caspase-6, and caspase-9. In accordance with recently reported results in vitro, these results indicate that activation of the PI3K/Akt/GSK3*β* signaling pathway by Sal protects against the neurotoxic conditions associated with the MPTP-induced PD mouse model.

## Abbreviations

PD: Parkinson's disease
DA: Dopaminergic
SNpc: Substantia nigra pars compacta
PI3K: Phosphoinositide 3-kinase
GSK-3: Glycogen synthase kinase-3
Sal: Salidroside
TH: Tyrosine hydroxylase
MPTP: 1-Methyl-4-phenyl-1,2,3,6-tetrahydropyridine
MPP^+^: 1-Methyl-4-phenyl-pyridinium
Bcl-2: B-cell lymphoma-2
Caspase: Cysteine-dependent aspartate-directed proteases.
